# Applicability of Novel Urinary Biomarkers for the Assessment of Renal Injury in Selected Occupational Groups in Sri Lanka: A Comparative Study with Conventional Markers

**DOI:** 10.3390/ijerph19095264

**Published:** 2022-04-26

**Authors:** E. M. D. V. Ekanayake, T. D. K. S. C. Gunasekara, P. Mangala C. S. De Silva, Sudheera Jayasinghe, E. P. S. Chandana, Nishad Jayasundara

**Affiliations:** 1 Department of Biomedical Sciences, School of Medicine and Health Sciences, University of North Dakota, Grand Forks, ND 58203, USA; emdvekanayake@gmail.com; 2 Department of Zoology, Faculty of Science, University of Ruhuna, Matara 81000, Sri Lanka; sameera.ac@live.com; 3 Department of Pharmacology, Faculty of Medicine, University of Ruhuna, Galle 80000, Sri Lanka; sudheerasj@yahoo.com; 4 Department of Biosystems Technology, Faculty of Technology, University of Ruhuna, Matara 81000, Sri Lanka; epschandana@zoo.ruh.ac.lk; 5 The Nicholas School of the Environment, Duke University, Durham, NC 27708, USA; nj58@duke.edu

**Keywords:** kidney diseases, biomarkers, early diagnosis, occupational groups, mass screening

## Abstract

Screening approaches with more robust biomarkers, are of the utmost importance in the characterization of renal injuries, particularly among communities with high burdens of chronic kidney disease of uncertain etiology (CKDu). The present study aimed to assess the utility of two emerging biomarkers: kidney injury molecule (KIM-1) and neutrophil gelatinase-associated lipocalin (NGAL) in predicting renal injury in different occupational groups in Sri Lanka. A cross-sectional study was conducted with six occupational groups (*n* = 188): fisherfolk (FF), paddy farmers (PF), sugarcane farmers (SF), factory workers (FW) and plantation workers (PW) to assess the predictive performance of KIM-1 and NGAL against a CKDu patient (PT) group (*n* = 40). The median KIM-1 levels of the study groups; FF, PF, SF, FW, PW and PT were 0.67, 0.59, 0.49, 1.62, 0.67 and 5.24 ng/mgCr, respectively, while the median NGAL levels were 1.16, 2.52, 1.42, 1.71, 1.06 and 22.41 ng/mgCr respectively. In ROC analysis to predict CKDu susceptibility, the area under the curve for KIM-1 ranged from 0.88 to 0.99 for the study groups, and in overall analysis, the sensitivity and specificity were 100% and 96%, respectively, for a cutoff value of 2.76 ng/mgCr. Similarly, for NGAL the range of AUC was 0.78–0.94, and a cutoff value of 3.12 ng/mgCr produced 88% sensitivity and 82% specificity. Compared with conventional markers, KIM-1 was the best biomarker for the characterization of renal injury in the participants of the occupational groups. With further validations, KIM-1 may be adopted as a prognostic marker to identify early renal injury and CKDu susceptibilities in community screening.

## 1. Introduction

Over the last few decades, a peculiar form of chronic kidney disease (CKD) has continued to emerge, predominantly in association with agricultural communities in hot humid tropics across the globe. This neophropathy is known as CKD of uncertain etiology (CKDu), and has become predominant in several global hotspots, including some countries in Central America: El Salvador, Nicaragua, Guatemala, Mexico, Panama, and Costa Rica [1], Sri Lanka [2], Andhra Pradesh in India, and the El-Minia Governorate in Egypt [3]. The Etiology behind CKDu in both Sri Lanka and Central America is debated, and the exact cause of this unique form of CKD is yet unknown [4]. A consistent histopathological and biochemical characteristics associated with the disease in its global hotspots, suggest a potentially common etiology [5]. According to epidemiological evidence, CKDu is predominant in rural agricultural communities and disproportionately affects young to middle-aged male farmers [6,7].

In the current clinical settings, community screening for the compromised renal function is mainly based on conventional markers such as dipstick proteinuria, urinary albumin to creatinine ratio (ACR) and serum creatinine (SCr) [2,8]. In a recent study on the prevalence of CKDu in Sri Lanka, conducted by the World Health Organization (WHO), ACR has been used as the leading diagnostic tool to identify susceptible cases [8]. Moreover, estimated Glomerular Filtration Rate (eGFR) is used in combination with ACR for defining CKD stages, and assessment of CKD risk. However, histopathological findings suggest that CKDu is a tubulointerstitial disease, hence the use of proteinuria and SCr is rather questionable in detecting early renal damage [9,10]. Particularly, the disease is relatively asymptotic in the early stages, with no detectable changes in urinary albumin excretion, SCr, or eGFR. A number of studies have rendered the limitations of sensitivity and specificity of the conventional markers in the diagnosis of CKDu in the early stages, and the reliance of renal screening on the conventional markers, may underestimate the prevalence of CKDu within communities [11,12,13].

Several emerging urinary biomarkers with enhanced sensitivity and specificity, such as Kidney Injury Molecule-1 (KIM-1), Neutrophil Gelatinase-Associated Lipocalin (NGAL), N-acetyl beta glucosaminidase (NAG), Interleukin 18 (IL-18), Insulin-like Growth Factor-Binding Protein 7 (IGFBP7) and Tissue Inhibitor of Metalloproteinase-2 (TIMP-2) are preferably used for the detection of Acute Kidney Injury (AKI) [14]. The utility of such biomarkers in the characterization of nephropathies associated with CKDs is evident in communities impacted by Mesoamerican nephropathy [15,16]. Notably, while KIM-1 and NGAL remain more widely used in higher income countries, clinical use of these markers remain novel in the clinical settings of low-middle income countries. Recently, a few comparative studies in Sri Lanka have demonstrated the applicability of several novel biomarkers along with conventional markers in CKDu screening [11,12,17]. To date a comprehensive assessment of novel biomarkers in communities with diverse occupational and socioeconomic strata in different climatic zones has not been performed in Sri Lanka. Hence, the applicability and usefulness of these novel biomarkers or biomarker combinations have to be further validated through community studies to implement these biomarkers in the clinical practice. The main objective of the study was to perform a comparative assessment on the applicability of two novel urinary biomarkers, KIM-1 and NGAL, in predicting renal injury in different occupational communities in Sri Lanka. Furthermore, we aimed to compare the performance of these biomarkers in terms of sensitivity and specificity against the conventional markers used for the assessment of renal function in the current clinical practice.

## 2. Materials and Methods

### 2.1. Study Design and Participants

**Figure 1:**
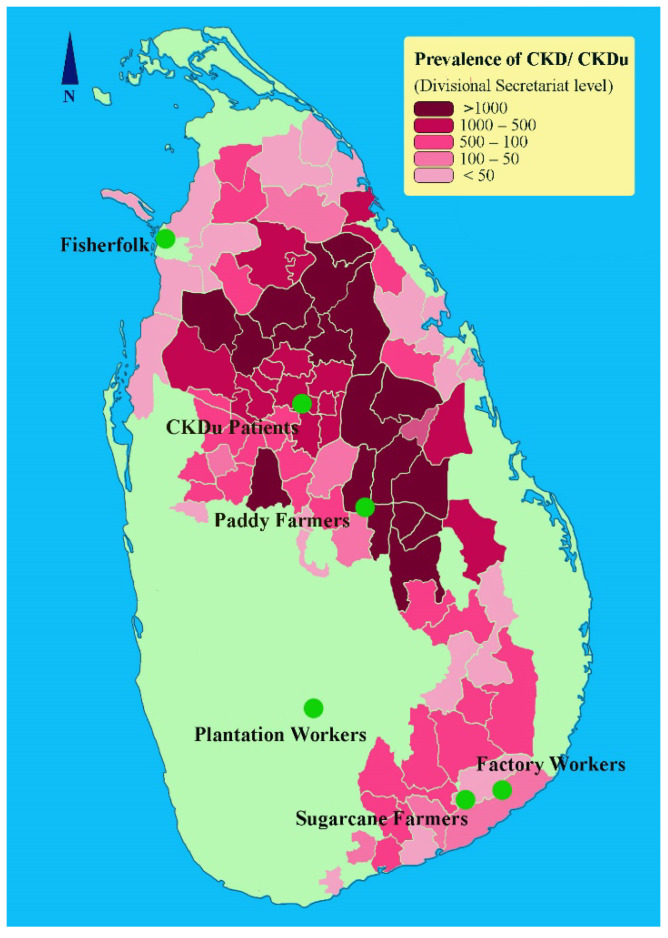
CKDu burden in Sri Lanka and the study locations. The prevalence of CKD/CKDu is given as the number of reported cases at the Divisional Secretariat level (Adapted from [7]).

A cross-sectional study was performed with five different occupational groups including fisherfolk (Mannar, Northern Province), sugarcane farmers and sugar factory workers (Buttala, Uva Province), paddy farmers (Wasgamuwa, North Central Province) and tea plantation workers (Nuwara Eliya, Central Province) along with a patient group diagnosed with CKDu (Anuradhapura, North Central Province) in Sri Lanka (Figure 1). Here, we considered CKDu patient group as the positive control as they had been clinically diagnosed with CKDu, and the subjects were in CKD stages 3, 4, and 5.

For the recruitment of participants, the minimum sample size was determined using the formula *n* = [(z^2^) P (1 − P)]/d^2^. The standard normal variate (z^2^) was taken as 1.96 at 5% type 1 error (*p* < 0.01) and the absolute error (d) was assumed to be 5% (d = 0.05). The prevalence of CKD (P) was taken as 2.35% for CKDu endemic regions in North Central Province, based on the most recent study by Ranasinghe et al. [7]. As per the calculations from the above formula, the minimum sample size was 36 for the regions with the highest burden of CKDu. The same number of participants were recruited for each occupational group. In the study, individuals involved in the selected occupations, for a consecutive period of 10 years, and clinically confirmed CKDu patients at CKD stages 3, 4 and 5, were eligible for participation. The selection of participants was performed based on systematic random sampling using the electoral register in each Grama Niladhari (GN) division. The eligible individuals in all occupational groups and the patient group were ranked according to their age with a sequential number assigned for each individual. Using a computer-generated random number table, 33–40 individuals from each study group were selected for the assessment of renal biomarkers.

### 2.2. Data and Sample Collection

An interviewer-administered pre-tested questionnaire was used for collecting demographic and socioeconomic data and details on health status, lifestyle and exposure to potential risk factors from the participants. An onsite medical examination was conducted, and resting blood pressure, height and weight of each participant were measured, in addition to the inspection of medical records. 

An early morning first void non-fasting urine sample and a non-fasting blood sample were obtained from each individual. Urine samples were collected into sterile containers (50 mL), while blood samples were collected into sterile serum separator tubes. Qualified medical professionals were involved in the medical examination and blood sampling. 

### 2.3. Sample Preparation and Analysis

Blood samples were kept standing for 30 min for coagulation. Following centrifugation at 1000× *g* for 15 min at 37 °C, serum was transferred into plain vacutainer tubes. Serum and urine samples were temporarily stored at 2–4 °C, and transported to the laboratory within 24 h. Processed samples were stored at −80 °C until analysis, and all the analyses were completed within five days.

Quantitative assessment of SCr, serum Cystatin-C (SCys-C), blood urea nitrogen (BUN), serum uric acid (SUA), urinary creatinine (UCr) and urinary microalbumin (UmALB) was performed with an automated biochemistry analyzer (Humasatr100, Human mbH, Wiesbaden, Germany) in the biochemistry laboratory of the Department of Zoology, Faculty of Science, University of Ruhuna, Sri Lanka. The instrument was calibrated for the mentioned biochemical assays using the standards and calibrators from the manufacturer before sample analysis along with quality control.

Urine samples were centrifuged at 1000× *g* for 10 min and the supernatant was taken for the analysis. Quantitative analysis of KIM-1 and NGAL in urine samples was performed with Enzyme-linked immunosorbent assay (ELISA) using the assay kits (CUSABIO, Wuhan, China) according to the manufacturer’s assay protocol. A microplate spectrophotometer (Epoch 2; Biotek Instruments, Winooski, VT, USA) was used for absorbance measurements. The detection ranges for KIM-1 and NGAL were 0.312–20 ng/mL and 15.6–1000 pg/mL respectively. 

### 2.4. Diagnostic Criteria

For the assessment of renal function, SCr, SCys-C, BUN, SUA, ACR and eGFR were used as the diagnostic criteria. Obesity was defined in terms of body mass index (BMI) ≥27 kg/m^2^ [18] and hypertension was defined as either systolic blood pressure ≥140 mmHg or diastolic blood pressure ≥90 mmHg, as confirmed with repeated measures [19]. Albuminuria was defined as ACR ≥30 mg/g. Based on the CKD-EPI creatinine-cystatin C equation (2021), eGFR was calculated [20].

### 2.5. Statistical Analysis

All the categorical variables were represented as numbers and percentages. Urinary KIM-1 and NGAL concentrations were adjusted to urinary creatinine before the data analysis. A Shapiro–Wilk test was used to assess the normality of clinical data of the study groups. Clinical data showed significant deviations from normality; hence a non-parametric approach was adopted for statistical analysis. Kruskal–Wallis one-way analysis followed by Dunn’s multiple comparison test was used for the comparison of clinical parameters among the study groups. Receiver-operator characteristic (ROC) analysis was performed to assess the diagnostic performance of the biomarkers. To construct ROC curves, the presence of CKDu was considered as the outcome variable. Each occupational group (negative outcome) was compared with the CKDu patient group (positive outcome). SCr, SCys-C, BUN, AUA, ACR, eGFR and creatinine-adjusted urinary KIM-1 and NGAL concentrations were used for the ROC analysis with no normalization or transformation. Multiple linear regression analysis was performed to characterize potential associations of KIM-1, NGAL, ACR and eGFR with several predictor variables: age, gender, body mass index (BMI), hypertension, diabetes mellitus, occupation, quality of drinking water source, agrochemical exposure, habits of smoking and liquor consumption. Statistical analyses and representations were conducted using GraphPad Prism 9.3 (GraphPad software LLC, San Diego, CA, USA), IBM SPSS Statistics 26.0 (IBM INC., New York, NY, USA) and NCSS 2021 (NCSS, LLC., Kaysville, UT, USA).

### 2.6. Ethical Considerations

The study was conducted in accordance with the declaration of Helsinki, under the approval of the Ethics Review Committee of the Faculty of Medicine, University of Ruhuna, Matara, Sri Lanka. (Reference No: 09.03.2016:3.2). Informed written consent was obtained from each participant before enrollment for the study. The participants granted consent for the provision of data and samples and the use of results for publications.

## 3. Results

### 3.1. Characteristics of the Study Participants

Table 1 presents the demography, health, and lifestyle data of the participants in the five occupational groups and the CKDu patient group. 

Diabetes mellitus and hypertension were the most common comorbidities observed among the participants, and the prevalence of these comorbidities was significantly higher among CKDu patients, compared to the other occupational groups. According to the medical history, at the time of diagnosis for CKDu, the patients had shown no evidence of diabetes mellitus or hypertension.

### 3.2. Novel Urinary Biomarkers

Table 2 presents the creatinine-adjusted urinary biomarkers KIM-1 and NGAL in the study participants.

**Figure 2:**
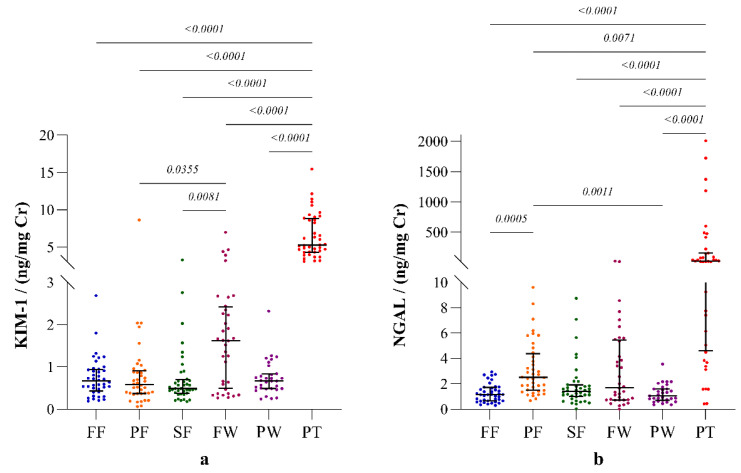
Biomarkers of renal injury: (**a**) kidney injury molecule (KIM-1); (**b**) neutrophil gelatinase-associated lipocalin (NGAL) in the study participants. The plots represent biomarker distribution with median and interquartile ranges. Inter-group statistical significance is shown with *p* values as implied by the Kruskal- Wallis test followed by Dunn’s multiple comparison test. Occupational groups: FF: fisherfolk, PF: paddy farmers, SF: sugarcane farmers, FW: factory workers, PW: plantation workers and PT: CKDu patients.

The highest expression of both KIM-1 and NGAL was observed in CKDu patients. The expression of both biomarkers showed substantial variation among the occupational groups (Figure 2).

Among the occupational groups, the highest urinary KIM-1 level was observed from sugar factory workers, and this was significantly higher than those of paddy and sugarcane farmers. On the contrary, paddy farmers showed the highest NGAL level, which was significantly higher than those of the fisherfolk and plantation workers. According to the interpretations of the clinical studies in the regions with no records of CKDu prevalence, and with least exposure to potential risk factors of CKDu according to observational studies in Sri Lanka, the mean (range) KIM-1 and NGAL levels of healthy individuals are known as 0.17 (0.0–3.4) ng/mg Cr and 0.47 (0.0–1.63), respectively [12]. The biomarker levels reported in all the study groups in our study are higher than those values of the healthy individuals with least exposure.

### 3.3. Conventional Markers

The group of the patients showed the highest levels of SCr, SCys-C, BUN and SUA along with the lowest eGFR (Table 3).

**Figure 3:**
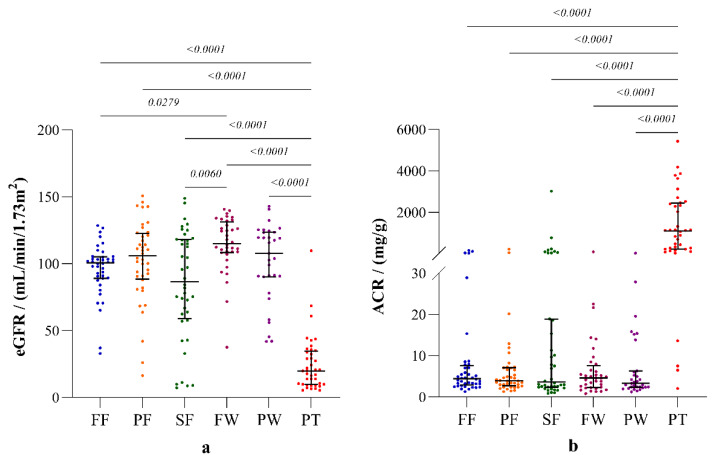
Parameters of renal function: (**a**) estimated glomerular filtration rate (eGFR) according to CKD-EPI creatinine-Cystatin-C equation 2021; (**b**) albumin creatinine ratio (ACR). The plots represent distribution with median and interquartile ranges. Inter-group statistical significance is shown with *p* values as implied by the Kruskal-Wallis test followed by Dunn’s multiple comparison test. Occupational groups: FF: fisherfolk, PF: paddy farmers, SF: sugarcane farmers, FW: sugar factory workers, PW: plantation workers, and PT: CKDu patients.

The incidence of albuminuria (ACR ≥ 30 mg/g) and declined eGFR (eGFR < 60 mL/min/1.73 m^2^) was at its highest among the sugarcane farmers, compared to the other occupational groups. The distributions of eGFR and ACR in the study groups are shown in Figure 3. 

Both clinical markers of renal function, eGFR and ACR, were significantly different (*p* < 0.0001) in the occupational groups compared to the patient group (Figure 3). Sugar factory workers reported the highest eGFR that was significantly different compared to those of the fisherfolk and sugarcane farmers. However, ACR showed no substantial differences among the occupational groups. 

**Figure 4:**
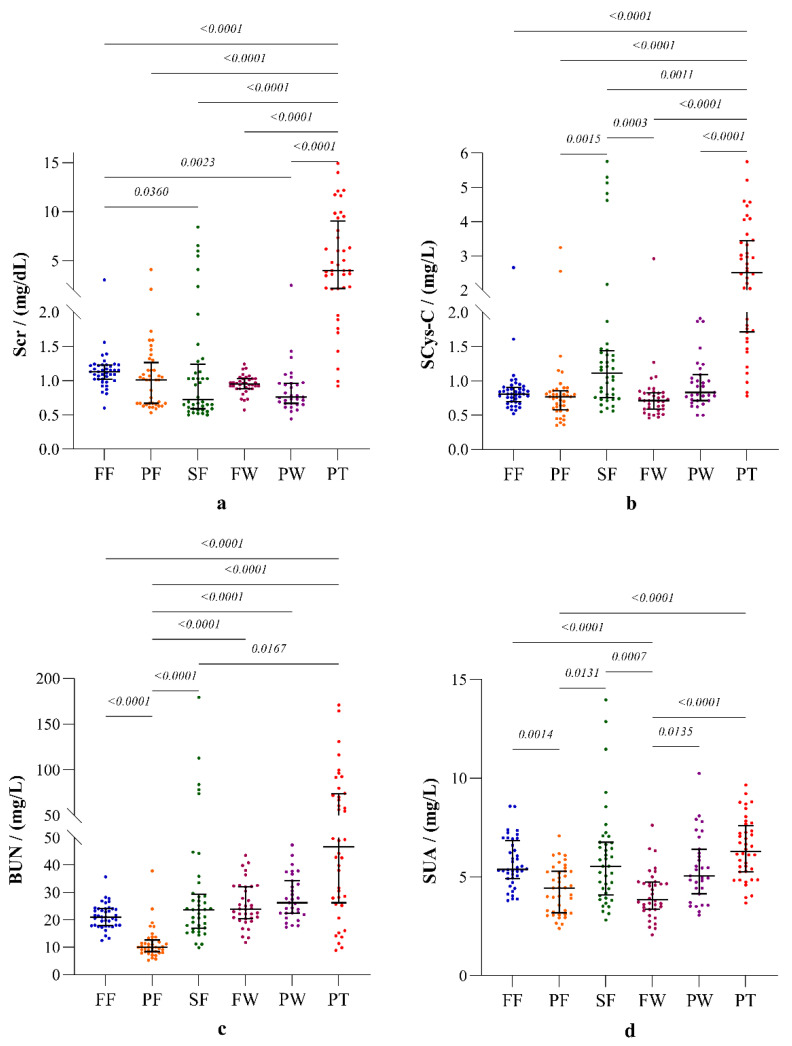
Parameters of renal function: (**a**) serum creatinine (SCr), (**b**) serum cystatin C (SCys-C), (**c**) blood urea nitrogen (BUN), and (**d**) serum uric acid (SUA). The plots represent distribution with median and interquartile ranges. Inter-group statistical significance is shown with *p* values as implied by the Kruskal-Wallis test followed by Dunn’s multiple comparison test. Occupational groups: FF: fisherfolk, PF: paddy farmers, SF: sugarcane farmers, FW: sugar factory workers, PW: plantation workers, and PT: CKDu patients.

Furthermore, the other four clinical markers: SCr, SCys-C, BUN and SUA also showed substantial differences among the study groups (Figure 4).

### 3.4. Assessment of Biomarker Performance

**Figure 5:**
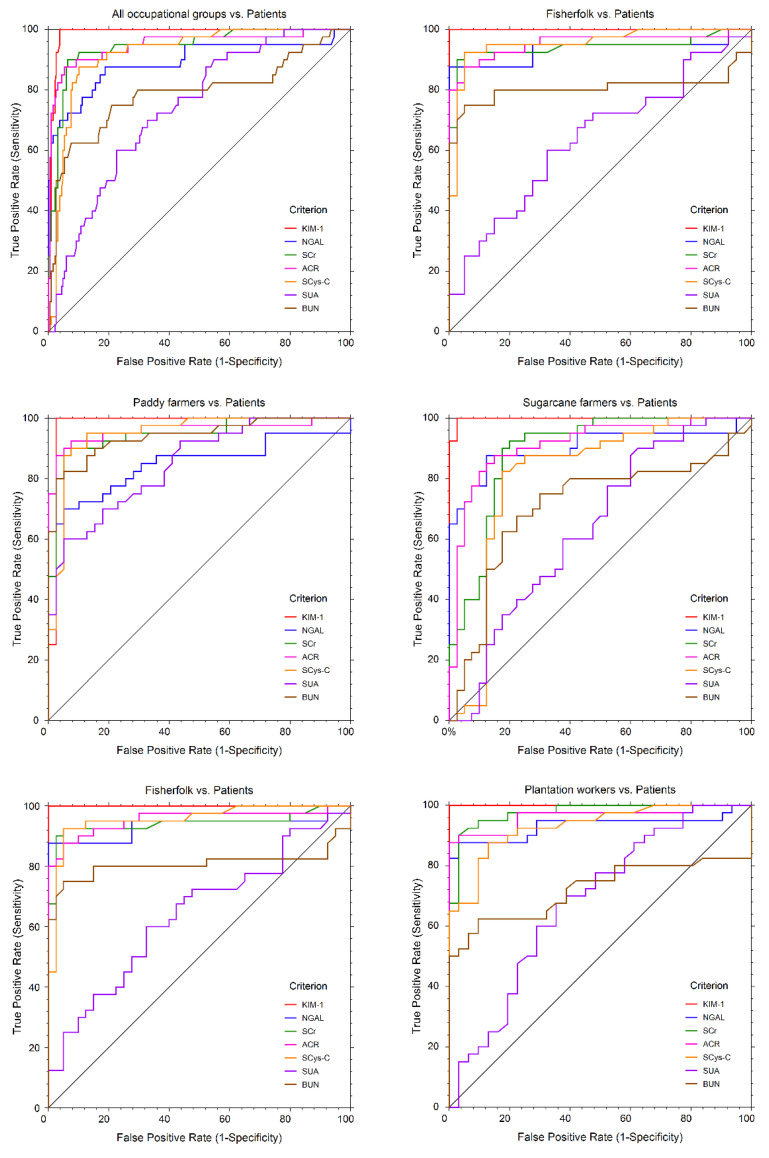
Receiver Operator Characteristic (ROC) curves of conventional and novel urinary biomarkers. SCr: serum creatinine, SCys-C: serum cystatin C, ACR: albumin creatinine ratio, BUN: blood urea nitrogen, SUA: serum uric acid, KIM-1: kidney injury molecule, NGAL: neutrophil gelatinase-associated lipocalin.

ROC curves for the assessment of biomarker performance in the occupational groups in comparison to the patients are shown in Figure 5. 

In the overall analysis, KIM-1, SCr, ACR and SCys-C produced excellent classifier models with area under the curve (AUC) values above 0.9. Based on the sensitivity and specificity of the biomarkers in the classifier models, KIM-1 served as the most robust biomarker in the characterization of renal injury (Table 4). 

However, in comparison to all biomarkers, KIM-1 produced a cut-off value of 2.76 ng/mgCr with 100% sensitivity and 96% specificity in predicting the susceptibility of CKDu among the studied communities.

Multiple linear regression analysis revealed significant associations of several variables with the urinary expression of KIM-1 and NGAL, and the two conventional markers, ACR and eGFR (Table 5).

Among the predictor variables, occupation showed significant associations with the four markers, while the effect of diabetes mellitus was significant only for KIM-1 and NGAL.

## 4. Discussion

The expression of both novel biomarkers, KIM-1 and NGAL, was significantly higher in CKDu patients, as expected. The declined renal function of the patients was further evidenced by significantly elevated ACR and significantly declined eGFR, compared to the other occupational groups. The distributions of biomarkers, except ACR showed significant variation among the occupational groups to varying degrees. In comparison to the other occupational groups, the proportions of albuminuria (ACR ≥ 30 mg/g) and declined eGFR (eGFR < 60 mL/min/1.73 m^2^) were at the highest among sugarcane farmers, with significant differences compared to the other occupational groups. Furthermore, the highest median levels of SUA, SCys-C, and the lowest median of eGFR were observed among sugarcane farmers with substantial differences compared to the other study groups. However, there were no significant differences in ACR among the study groups. ROC analysis of biomarkers demonstrated KIM-1, SCr, ACR, and SCys-C as potential biomarkers in predicting renal injury, with AUC values above 0.9. In terms of sensitivity and specificity, KIM-1 performed better in the prediction. In the classifier model for KIM-1, AUC was 0.988 while sensitivity and specificity were 100% and 96%, respectively, for a cutoff value of 2.76 ng/mgCr. Although a novel biomarker, the predictive power of NGAL was not as significant as KIM-1 in the study communities.

Emerging evidence from recent research suggests certain novel biomarkers including KIM-1 and NGAL as more robust indicators of renal injury in comparison to the conventional markers, particularly in community screening in CKDu-affected regions. The applicability of these novel biomarkers in the settings of Mesoamerican nephropathy is much more evident [6,21]. However, the utility of these biomarkers, particularly in the characterization of early renal injury in CKDu-affected communities in Sri Lanka has not been well studied. Hence, the main focus of the present study was to assess the applicability of KIM-1 and NGAL for the early diagnosis of renal injury in selected occupational groups in Sri Lanka, along with a patient group diagnosed with CKDu. According to the interpretations on albuminuria and declined eGFR, there may be a high incidence of potential renal damage among sugarcane farmers. However, this observation is not always supported by other biomarkers, as they do not exhibit distinct differences compared to all other occupational groups. Furthermore, the two novel biomarkers, KIM-1 and NGAL, did not exhibit profound differences among the occupational groups. Hence, within the context of these findings, it is difficult to reliably identify a particular occupational group with a high risk of compromised renal function, mainly due to the low sample size. Importantly, our main focus was to assess the applicability of the novel biomarkers, KIM-1 and NGAL, against the conventional markers for the identification of renal damage in different occupational groups. In that context, we identified KIM-1 as the best marker to indicate renal injury over the other biomarkers, based on ROC analysis. 

A recent analysis of biomarker studies rendered the utility of several novel biomarkers, including KIM-1 and NGAL, to characterize early renal injury in adult and pediatric communities in Mesoamerica and Sri Lanka [4]. In 2016, De Silva et al. [12] demonstrated the potential utility of urinary KIM-1 and NGAL for detecting early renal damage among farming communities in Sri Lanka, in the absence of albuminuria. This study indicated that creatinine-adjusted urinary KIM-1 has some potential to diagnose early CKDu cases over the other conventional and novel biomarkers. This was further confirmed with the high sensitivity and specificity of KIM-1 in three ROC models, including sugarcane workers and paddy farmers in regions with emerging evidence of CKDu [12]. On the contrary, Wanigasuriya et al. (2018) demonstrated that specificities of Cys-C, KIM-1 and clusterin were very low for given sensitivities when comparing healthy individuals even with diagnosed CKDu patients at stage 3 or above. Hence, the researchers did not identify KIM-1 as an effective biomarker for screening tests [11]. However, consistent with our findings, the utility of KIM-1 in combination with two other biomarkers, alpha-1-microglobulin (α1M) and retinol-binding protein-4 (RBP-4), to differentiate CKD cases from healthy individuals was shown by Fernando et al. [21], with an overall sensitivity of ≥0.867 and specificity ≥0.765. Furthermore, a combination of KIM-1 with osteopontin and RBP-4 was shown to be capable of distinguishing CKDu patients from CKD patients with both a sensitivity and specificity of ≥0.93. The role of KIM-1 and NGAL as indicators of potential renal injury has also been studied in association with Mesoamerican communities. Multiple studies have shown that significantly elevated urinary expression of KIM-1 and NGAL in sugarcane field workers along with elevated levels of SCr and BUN and reduced eGFR during the work shifts [4]. Furthermore, elevated expression of KIM-1 and NGAL has been observed in children and adolescents from regions with a high burden of CKDu in Nicaragua [22] and El Salvador [23]. These studies provide evidence that proves the potential utility of KIM-1 and NGAL as prognostic indicators of renal injury and susceptibility of CKDs. However, an assessment of the diagnostic performance of these biomarkers in terms of sensitivity and specificity has not been performed in a Mesoamerican context. 

There are several noteworthy strengths in the present study. In clinical practice, community screening for declined kidney function, and the identification of susceptible cases is mainly based on dipstick proteinuria, ACR, and SCr [2,8]. However, the accuracy and reliability of such conventional markers are often questioned. A few comparative studies using conventional markers and novel markers in CKDu screening have been recently reported [11,12,17]. However, a detailed comparison of different occupational groups and inhabitants in different climatic zones does not exist. Furthermore, the effect of currently known risk factors of CKDu, such as agrochemical exposure, heat exposure, contaminated drinking water, climatic factors, and food and lifestyle habits, on individuals varies depending on the occupation and residential climatic zones. Therefore, this study was focused on several occupational groups (fishermen, paddy farmers, sugarcane factory workers, sugarcane farmers and Plantation workers) from the three climatic zones (arid zone, dry zone and wet zone) in Sri Lanka, and it aimed to find the most specific and sensitive marker for early CKDu diagnosis. According to the findings, KIM-1 appeared to be the best marker to identify compromised renal function in the study groups.

In this study, CKDu patient group was considered as the positive control where all the individuals were confirmed CKDu subjects at CKD stages 3, 4, and 5. It is known that CKDu is different from CKD, and this nephropathy is not associated with diabetes, glomerulonephritis, hypertensive renal diseases, and polycystic kidneys [2,16,24,25,26]. However, here we found 52.5% of patients with diabetes and 30% of patients with hypertension in the CKDu patient group. This could be expected in the selected individuals were with end-stage kidney disease. Importantly, past medical history records of all patients in the CKDu patient group confirmed the absence of diabetes or hypertension at the time of diagnosis. Moreover, a significant association of occupation with KIM-1, NGAL, ACR, and eGFR was observed in regression analysis. This may indicate certain effects of the nature of occupation on renal function. However, several factors, such as environmental toxins, heat stress and dehydration tendencies, level of physical activity, and lifestyle, potentially affect renal health while the characterization of such associations with detailed studies is an important measure to ensure occupational renal health. 

In addition, the present study presents several limitations. The main limitation is the low sample size, although we have met the required minimum sample size for the study as calculated by a power analysis. The lack of a negative control group also appears to be a limitation of our study. Depending on the occupation lifestyle, contamination of food and water with potential nephrotoxic agents, communities are likely exposed to potential environmental risk factors. For instance, fishermen are more exposed to heat compared to the farmers and, on the contrary, farmers are more exposed to agrochemicals in farming fields while fishermen do not experience agrochemical exposure. Furthermore, the climate is also different from region to region. Studies indicate that several environmental risk factors such, as heavy metals, agrochemicals, fluoride and hardness in drinking water, heat exposure and dehydration tendencies, and environmental toxins, serve as the main risk factors for renal diseases including CKDu [6], but their role may vary across communities in the country. Hence, the selection of an ideal negative control group is challenging. However, a recent clinical study in communities from the regions with no evidence of CKDu incidence and known to have the lowest level of exposure to the potential risk factors reported medial KIM-1 and NGAL levels comparatively lower than those of the occupational groups participated in the present study.

In our study, the main focus was not to assess the prevalence of CKDu susceptibility but to assess the performance of KIM-1 and NGAL in predicting renal damage and abnormal renal function in diverse occupational groups in Sri Lanka. Here, we have shown a better performance of KIM-1 over the other biomarkers in predicting abnormal renal function. However, as the sample size is the main limitation of our study, the prognostic utility of KIM-1 requires further validation with detailed studies. Furthermore, there are no established cutoff values for KIM-1 and NGAL to distinguish CKD/CKDu cases from healthy individuals in Sri Lanka. The sensitivity and specificity of KIM-1 and NGAL over conventional markers to predict early renal injury has been shown in many studies. The establishment of cutoff values is a critical milestone in the journey of these novel biomarkers to the level of clinical biomarkers of renal injury, from the experimental level. Hence these biomarkers should be further validated, and we recommend in-depth studies in association with different age and occupational groups in different clinical settings.

## 5. Conclusions

Common conventional markers such as SCr, SCysC, and ACR had low specificity and sensitivity, and hence were not suitable for the early diagnosis of renal injury. According to our analysis, creatinine adjusted novel urinary biomarker KIM-1 had the highest sensitivity and specificity for detecting early renal injury. However, here we have produced preliminary findings, and it requires further validation with detailed studies with an increased sample size. Moreover, with further validations, KIM-1 may be developed as a robust clinical tool to diagnose abnormal renal function and disease susceptibilities in mass screening.

## Figures and Tables

**Table 1:** Demographic and clinical characteristics of the study participants.

Characteristics	Fisherfolk (*n* = 40)	Paddy Farmers (*n* = 40)	Sugarcane Farmers (*n* = 40)	Factory Workers (*n* = 33)	Plantation Workers (*n* = 35)	CKDu Patients (*n* = 40)
**Demographic characteristics**						
**Age/years ^†^**	47 (37–54)	46 (32–55)	50 (36–62)	37 (29–46)	47 (39–56)	53 (47–65)
**Gender** Male Female	27 (67.5) 13 (32.5)	12 (30.0) 19 (70.0)	24 (60.0) 16 (40.0)	21 (63.6) 12 (36.4)	16 (45.7) 19 (54.3)	26 (65.0) 14 (35.0)
**Clinical characteristics**						
**BMI/kgm^−2 †^**	23.7 (22.1–26.7)	21.7 (19.4–25.6)	20.2 (17.3–22.9)	19.9 (18.6–22.6)	20.8 (18.4–24.6)	23.5 (22.0–26.0)
**Obesity ^⁑^**	5 (12.5)	5 (12.5)	1 (2.5)	1 (3.0)	2 (5.7)	1 (2.5)
**Diabetes mellitus ^⁑^**	5 (12.5) *	3 (7.5) *	0 *	0 *	2 (5.7) *	21 (52.5)
**Hypertension ^⁑^**	6 (15.0) *	3 (7.5) *	5 (12.5) *	0 *	4 (11.4) *	12 (30.0)
**Lifestyle-related characteristics**						
**Agrochemical Exposure ^⁑^**	0	40 (100.0)	32 (80.0)	0	22 (62.9)	28 (70.0)
**Drinking water quality** ^⁑^ Low Medium High	0 29 (72.5) 11 (27.5)	20 (50.0) 19 (47.5) 21 (52.5)	6 (15.0) 34 (85.0) 6 (15.0)	2 (6.0) 22 (66.7) 11 (33.3)	4 (11.4) 31 (88.6) 0	0 28 (70.0) 12 (30.0)
**Smoking ^⁑^**	6 (15.0)	2 (5.0)	18 (45.0)	17 (51.2)	15 (42.9)	1 (2.5)
**Alcohol consumption ^⁑^**	19 (47.5)	10 (25.0)	15 (37.5)	17 (51.5)	22 (42.9)	3 (2.5)

**^†^** Values are expressed as median (interquartile distance). * Denotes statistical significance compared to the patient group. ^⁑^ The incidence is given as the number of cases (percentage) for each study group and the statistical significance is expressed as implied by the Chi-squared test. Obesity is expressed in terms of body mass index (BMI) ≥27 kg/m^2^. Drinking water quality, Low: untreated water from shallow surface wells, Medium: disinfected water from water treatment plants, High: bottled water, filtered water from domestic filters or reverse osmosis (RO) filters. Lifestyle characteristics are based on self-reported data.

**Table 2:** Creatinine-adjusted biomarker levels.

Study Group	KIM-1 (ng/mg Cr)	NGAL (ng/mg Cr)
Fisherfolk	0.665 (0.428–0.945)	1.155 (0.653–1.715)
Paddy farmers	0.585 (0.373–0.910)	2.515 (1.498–4.380)
Sugarcane farmers	0.490 (0.383–0.703)	1.420 (0.998–1.910)
factory workers	1.625 (0.500–2.418)	1.710 (0.733–5.470)
Plantation workers	0.67 (0.490–0.840)	1.060 (0.690–1.600)
CKDu patients	5.242 (4.298–8.800)	22.41 (4.630–154.300)

Urinary biomarker levels are given as median (inter quartile distance). KIM-1: Kidney injury molecule; NGAL: Neutrophil gelatinase-associated lipocalin.

**Table 3:** Creatinine-adjusted biomarker levels.

Clinical Parameters	Fisherfolk (*n* = 40)	Paddy Farmers (*n* = 40)	Sugarcane Farmers (*n* = 40)	Factory Workers (*n* = 33)	Plantation Workers (*n* = 35)	CKDu Patients (*n* = 40)
**ACR (mg/g)**	4.455 (2.885–7.645)	3.945 (2.755–7.110)	3.640 (2.415–18.88)	4.600 (2.323–7.613)	3.350 (2.430–6.350)	1105.00 (227–2458)
**eGFR** **(mL/min/1.73 m^2^)**	100.7 (89.1–105.3)	106.0 (88.5–122.7)	86.7 (59.0–118.2)	115.1 (108.4–131.3)	107.7 (90.3–123.5)	20.0 (9.9–34.8)
**SCr (mg/dL)**	1.130 (1.02–1.228)	1.010 (0.670–1.263)	0.725 (0.590–1.243)	0.950 (0.880–1.030)	0.760 (0.670–0.960)	3.990 (2.150–9.035)
**SCys-C (mg/L)**	0.810 (0.693–0.903)	0.770 (0.578–0.855)	1.110 (0.760–1.440)	0.710 (0.590–0.828)	0.830 (0.710–1.090)	2.510 (1.715–3.443)
**BUN (mg/dL)**	21.00 (17.95–24.10)	10.00 (8.40–12.70)	23.70 (16.95–29.45)	23.90 (20.48–32.15)	26.30 (22.50–34.30)	46.65 (26.28–73.73)
**SUA (mg/dL)**	5.385 (4.925–6.850)	4.445 (3.185–5.288)	5.545 (4.090–6.753)	3.850 (3.353–4.745)	5.060 (4.140–6.410)	6.295 (5.258–7.593)
**ACR ≥ 30 mg/g**	3 (7.5) ^†,^*	2 (5.0) ^†,^*	9 (22.5) ^†^	1 (3.0) ^†,^*	1 (2.9) ^†,^*	40 (100)
**eGFR < 60 mL/min/1.73 m^2^**	2 (5.0) ^†,^*	3 (7.5) ^†,^*	10 (25.0) ^†^	1 (3.0) ^†,^*	5 (14.3) ^†^	40 (100)

Estimated Glomerular Filtration Rate (eGFR) is given according to the CKD-EPI creatinine-cystatin C equation (2021). Clinical parameters are expressed as median (interquartile distance) for each group. The incidence of elevated ACR and declined eGFR are given as the number of cases along with its proportion of the total size of the respective occupational group and the statistical significance is expressed as implied by the Chi-squared test. ^†^ Significant compared to the patient group (*p* < 0.0001); * significant compared to the sugarcane farmers (*p* < 0.05). ACR: albumin creatinine ratio, SCr: serum creatinine, SCys-C: serum cystatin C, BUN: blood urea nitrogen, SUA: serum uric acid.

**Table 4:** Diagnostic performance of biomarkers.

Parameter	ROC Parameters	FF vs. PT	SW vs. PT	SF vs. PT	PF vs. PT	EW vs. PT	Overall
SCr (mg/dL)	AUC (95% CI)	0.942 (0.88–1.00)	0.977 (0.94–1.01)	0.891 (0.82–0.97)	0.947 (0.89–0.99)	0.978 (0.95–1.00)	0.944 (0.90–0.98)
	Cutoff	1.41	1.30	1.38	1.65	1.14	1.21
	Sensitivity	0.92	0.93	0.92	0.90	0.95	0.93
	Specificity	0.95	1.00	0.80	0.93	0.94	0.81
SCys-C (mg/L)	AUC (95% CI)	0.955 (0.91–1.00)	0.985 (0.97–1.00)	0.807 (0.70–0.91)	0.95 (0.90–0.99)	0.935 (0.88–0.99)	0.920 (0.88–0.96)
	Cutoff	0.98	1.09	1.42	0.97	1.11	1.09
	Sensitivity	0.95	0.93	0.88	0.95	0.92	0.93
	Specificity	0.88	0.97	0.75	0.88	0.80	80.7
ACR (mg/g)	AUC (95% CI)	0.954 (0.90–1.00)	0.963 (0.92–1.07)	0.926 (0.87–0.98)	0.959 (0.91–1.00)	0.96 (0.91–1.01)	0.949 (0.90–0.99)
	Cutoff	8.64	12.71	28.21	13.28	10.60	8.55
	Sensitivity	0.92	0.92	0.90	0.92	0.92	0.93
	Specificity	0.83	0.88	0.78	0.93	0.80	0.77
BUN (mg/dL)	AUC (95% CI)	0.807 (0.69–0.92)	0.757 (0.64–0.87)	0.707 (0.59–0.83)	0.942 (0.89–0.99)	0.731 (0.61–0.85)	0.789 (0.69–0.89)
	Cutoff	20.85	37.25	37.00	13.55	38	24.5
	Sensitivity	0.80	0.62	0.62	0.92	0.62	0.8
	Specificity	0.48	0.88	0.83	0.80	0.91	0.69
SUA (mg/dL)	AUC (95% CI)	0.639 (0.52–0.76)	0.904 (0.84–0.97)	0.637 (0.52–0.76)	0.851 (0.77–0.93)	0.688 (0.57–0.81)	0.738 (0.66–0.82)
	Cutoff	6.94	5.37	7.00	5.79	6.41	4.76
	Sensitivity	0.38	0.72	0.38	0.62	0.50	0.90
	Specificity	0.78	0.91	0.78	0.88	0.80	0.45
KIM-1 (ng/mgCr)	AUC (95% CI)	0.998 (0.99–1.00)	0.910 (0.84–0.98)	0.875 (0.79–0.97)	0.975 (0.93–1.02)	0.999 (0.99–1.00)	0.988 (0.98–1.00)
	Cutoff	2.608	3.352	2.984	2.928	2.291	2.76
	Sensitivity	1.00	0.92	1.00	1.00	1.00	1.00
	Specificity	0.98	0.76	0.78	0.95	0.97	0.96
NGAL (ng/mgCr)	AUC (95% CI)	0.936 (0.87–1.00)	0.777 (0.67–0.88)	0.923 (0.86–0.99)	0.819 (0.72–0.92)	0.863 (0.78–0.95)	0.893 (0.82–0.96)
	Cutoff	1.54	10.88	1.59	7.58	7.36	3.12
	Sensitivity	0.95	0.12	0.90	0.68	0.70	0.88
	Specificity	0.75	0.88	0.75	0.93	0.86	0.82

SCr: serum creatinine, SCys-C: serum cystatin C, ACR: albumin creatinine ratio, BUN: blood urea nitrogen, SUA: serum uric acid, KIM-1: kidney injury molecule, NGAL: neutrophil gelatinase-associated lipocalin. Occupational groups; FF: fisherfolk, PF: paddy farmers, SF: sugarcane farmers, FW: sugar factory workers, PW: plantation workers, and PT: CKDu patients.

**Table 5:** Novel and conventional markers of renal function and effect of variables.

Predictor Variable	KIM-1		NGAL		ACR		eGFR	
	β	p	β	p	β	p	β	p
Age	−0.048	0.354	−0.094	0.174	−0.069	0.255	−0.163	0.002
Gender	0.06	0.264	−0.003	0.967	−0.32	0.612	−0.04	0.491
BMI	0.032	0.535	−0.05	0.462	0.041	0.494	0.012	0.829
Occupation	**0.415**	**<0.0001**	**0.207**	**0.002**	**0.292**	**<0.0001**	**−2.08**	**<0.0001**
Hypertension	**0.259**	**<0.0001**	0.093	0.272	**0.368**	**<0.0001**	**−0.487**	**<0.0001**
Diabetes mellitus	**0.182**	**0.001**	**0.233**	**<0.0001**	0.085	0.226	0.014	0.827
Quality of Drinking water	−0.05	0.385	−0.029	0.707	0.008	0.905	0.039	0.535
Agrochemical exposure	−0.09	0.881	−0.078	0.339	0.007	0.922	0.079	0.224
Smoking	−0.073	0.163	−0.06	0.392	**−0.137**	**0.015**	0.045	0.43
Alcohol consumption	**0.157**	**0.001**	0.091	0.216	−0.064	0.324	0.097	0.099

Predictor variables with significant association with biomarkers are given in bold. β: standard coefficient; p: probability; BMI: body mass index. ACR: albumin creatinine ratio, KIM-1: kidney injury molecule, NGAL: neutrophil gelatinase-associated lipocalin, and eGFR: estimated glomerular filtration rate.

## Data Availability

The data presented in this study are not publicly available due to the restrictions under ethical liabilities with the participants.
